# Air Pollution and Pulmonary Tuberculosis: A Nested Case–Control Study among Members of a Northern California Health Plan

**DOI:** 10.1289/ehp.1408166

**Published:** 2016-02-09

**Authors:** Geneé S. Smith, Stephen K. Van Den Eeden, Cynthia Garcia, Jun Shan, Roger Baxter, Amy H. Herring, David B. Richardson, Annelies Van Rie, Michael Emch, Marilie D. Gammon

**Affiliations:** 1 Department of Epidemiology, University of North Carolina, Chapel Hill, Chapel Hill, North Carolina, USA; 2 Division of Research, Kaiser Permanente Northern California, Oakland, California, USA; 3 California Air Resources Board, Sacramento, California, USA; 4 Department of Biostatistics,; 5 Carolina Population Center, and; 6 Department of Geography, University of North Carolina, Chapel Hill, Chapel Hill, North Carolina, USA

## Abstract

**Background:**

Ecologic analyses, case–case comparisons, and animal experiments suggest positive associations between air pollution and tuberculosis.

**Objectives:**

We evaluated this hypothesis in a large sample, which yielded results that are applicable to the general population.

**Methods:**

We conducted a case–control study nested within a cohort of Kaiser Permanente of Northern California members. All active pulmonary tuberculosis (TB) cases newly diagnosed between 1996 and 2010 (n = 2,309) were matched to two controls (n = 4,604) by age, sex, and race/ethnicity on the index date corresponding with the case diagnosis date. Average individual-level concentrations of carbon monoxide (CO), nitrogen dioxide (NO2), sulfur dioxide (SO2), ozone (O3), and particulate matter with aerodynamic diameter ≤ 2.5 μm (PM2.5) and 10 μm (PM10) for 2 years before diagnosis/entry into the study were estimated using measurements from the California Air Resources Board monitor closest to the participant’s residence.

**Results:**

In single-pollutant adjusted conditional logistic regression models, the pulmonary TB odds ratios (95% confidence intervals) for the highest quintile (vs. lowest) were 1.50 (95% CI: 1.15, 1.95) for CO and 1.42 (95% CI: 1.10, 1.84) for NO2. Corresponding estimates were higher among never [1.68 (95% CI: 1.26, 2.24)] than ever [1.19 (95% CI: 0.74, 1.92)] smokers for CO. In contrast, for NO2, estimates were higher among ever [1.81 (95% CI: 1.13, 2.91)] than never [1.29 (95% CI: 0.97, 1.71)] smokers. O3 was inversely associated for smokers [0.66 (95% CI: 0.43, 1.02)] and never smokers [0.65 (95% CI: 0.52, 0.81)]. No other consistent patterns were observed.

**Conclusions:**

In this first, to our knowledge, U.S. nested case–control study on air pollution and pulmonary TB, we observed positive associations with ambient CO and NO2, which require confirmation.

**Citation:**

Smith GS, Van Den Eeden SK, Garcia C, Shan J, Baxter R, Herring AH, Richardson DB, Van Rie A, Emch M, Gammon MD. 2016. Air pollution and pulmonary tuberculosis: a nested case-control study among members of a Northern California health plan. Environ Health Perspect 124:761–768; http://dx.doi.org/10.1289/ehp.1408166

## Introduction

Air pollution is a substantial cause of morbidity and mortality worldwide, resulting in major public health impacts and millions of dollars lost each year [Holgate et al. 1999; World Health Organization (WHO) 2011]. Recent meta-analyses have revealed an association between indoor air pollution, primarily from biomass fuel combustion, and tuberculosis (TB) disease (Lin et al. 2007; Sumpter and Chandramohan 2013). Ecologic studies, including several conducted in the United States, also suggest that TB is associated with ambient air pollution, including both short-term (Shilova and Glumnaia 2004) and long-term exposures (Iwai et al. 2005; Smith et al. 2014; Tremblay 2007). An ecologic study in Japan reported a correlation between annual total suspended particles in air and TB (Iwai et al. 2005). While exploring seasonal fluctuations of TB incidence in an ecologic analysis in Russia, Shilova and Glumnaia (2004) found that along with climatic factors, atmospheric pollutants [including nitric oxide (NO), carbon monoxide (CO), and sulfur dioxide (SO_2_)] were associated with TB incidence. In a recent study of 196 patients from two Los Angeles, California, hospitals, a correlation was observed between annual small particle-size particulate matter (PM_2.5_) and acid-fast bacilli (AFB) smear–positive TB compared with AFB-negative TB (Jassal et al. 2013). However, epidemiologic studies that consider a wide variety of individual-level air pollutant exposures in a large sample that is generalizable to the general population are lacking.

*Mycobacterium tuberculosis* is the causative agent of TB. The immune system is most often able to contain this infection; however, weakened immunity, caused by HIV, diabetes, and a host of other factors, can cause TB to reactivate (i.e., to progress from inactive to active infection) (Raviglione et al. 1995; WHO 2010). Biologically, air pollutants could be involved in the reactivation of TB through altering macrophage function, thereby increasing susceptibility to developing active TB. Tumor necrosis factor (TNF)-α and interferon-gamma (IFN-γ) play a central role in containing and inhibiting the growth of mycobacteria (Flad et al. 1995; Fremond et al. 2005), but animal experiments on the effects of air pollution on cytokine expression in general (Saito et al. 2002a) and in response to mycobacterial infection (Hiramatsu et al. 2005; Saito et al. 2002b) have both indicated decreased levels of these proteins. Long-term exposure to diesel exhaust has been shown to reduce TNF-α and IFN-γ production (Saito et al. 2002a, 2002b), to decrease expression of IFN-γ and inducible NO synthase mRNAs, and to increase the mycobacterial load in mice (Hiramatsu et al. 2005).

In this study, we aimed to investigate whether exposure to criteria air pollutants SO_2_, nitrogen dioxide (NO_2_), CO, ozone (O_3_), particulate matter with aerodynamic diameter of ≤ 10 μm (PM_10_), and particulate matter with aerodynamic diameter of ≤ 2.5 μm (PM_2.5_) is associated with increased risk of active pulmonary TB in a well-defined population of northern California residents.

## Methods

### Study Population

We conducted a nested case–control study of the association between air pollution and pulmonary TB disease among members of Kaiser Permanente Northern California (KPNC), an integrated health care delivery system providing care to 3.3 million residents in the greater San Francisco, Oakland, San Jose, Sacramento, and Fresno areas. KPNC serves ~25–30% of the entire population in the geographic areas served. Cases included all patients newly diagnosed with active pulmonary TB among adult KPNC members. Cases were individuals, between January 1996 and December 2010, with either: *a*) a new clinical diagnosis and a prescription for at least 30 days of anti-tuberculosis medication such as isoniazid, rifampin, ethambutol, or pyrazinamide; or *b*) an initial positive TB culture. For cases, diagnosis date, use of anti-TB drugs and relevant laboratory assays were abstracted from the KPNC clinical databases. Controls, selected from KPNC members free of TB on the index date of diagnosis of the case, were matched to cases (2:1) by age, sex, and race/ethnicity (non-Hispanic white, black, Asian, other). All cases and controls were KPNC members for a minimum of 2 years prior to entry into the study. Institutional Review Board approval was obtained from KPNC and from the University of North Carolina at Chapel Hill before research was initiated. This study was limited to analysis of existing data that involved no direct interaction with subjects and therefore met criteria for a waiver of informed consent.

### Exposure Assessment

Exposures estimates for each individual were determined using average ambient concentrations of PM_2.5_, PM_10_, O_3_, NO_2_, CO, and SO_2_ from all relevant monitors operating in California in the 24 months before diagnosis/entry into the study. Based on the assumption that air pollution acts to increase susceptibility to develop pulmonary TB upon exposure to *Mycobacterium tuberculosis*, we posited *a priori* that the etiologically relevant exposure window was the period within 24 months before the initial infection with TB (Raviglione et al. 1995; WHO 2010). Pollutant concentrations were obtained using monitoring stations from California’s State and Local Air Monitoring Network Plan (http://www.arb.ca.gov/aqd/netrpt/netrpt.htm) with ≥ 75% completeness in each month. Monitors with ≥ 25% incomplete data for a month were not included in the analysis; therefore, pollutant exposure measurements obtained from these monitors were recoded as missing and were not included in the analysis for that pollutant. The Interagency Monitoring of Protected Visual Environments (IMPROVE 2012) network, which provides additional coverage of PM_2.5_ in less-populated areas of the state, was included to supplement PM_2.5_ measurements because state and local agencies only began monitoring PM_2.5_ in 1999.

To assess individual-level air pollution exposure, geocoded patient home addresses (current and up to 2 years before diagnosis) were assigned the pollutant concentration of the closest available monitor (see Figures S1–S6 for the distribution of monitors in California). For each study participant, monthly exposures were estimated for each residence of record during the 24 months before diagnosis/entry into the study and were then added together to calculate the average of the aggregate exposures. In addition, we assigned average 8-hr values for CO and O_3_ {consistent with the values used for the National Ambient Air Quality Standards [U.S. Environmental Protection Agency (EPA) 2008b]} from these same monitors closest to each participant’s home address.

Potential individual-level confounders and effect measure modifiers were ascertained from KPNC’s electronic clinical databases, including data on age, sex, race/ethnicity, length of KPNC membership, cigarette smoking, alcohol hospitalization (defined as any record of hospitalization in which excessive alcohol intake/abuse was documented within the electronic clinical data), HIV status, comorbidity (diabetes, COPD, renal dialysis), and residential address history. To examine the potential role of smoking in the association between air pollution and pulmonary TB, a variable was constructed from all smoking information recorded in the KPNC electronic clinical database; if smoking was indicated anywhere in the records, the subject was considered an ever smoker; otherwise, the subject was considered a never smoker. Additionally, data from the 2000 U.S. Census were used to create block-level continuous variables (median household income, percent foreign-born, and percent with more than high school education) as indicators of socioeconomic status at diagnosis/study entry for factors not routinely collected at KPNC.

### Statistical Analysis

We used conditional logistic regression analysis, adjusting for all matching factors (age, sex, and race/ethnicity), to estimate the odds ratios (ORs) and 95% confidence intervals (CIs) for the association between pulmonary TB and each average air pollutant concentration (SO_2_, NO_2_, CO, O_3_, PM_10_ and PM_2.5_) assigned to each individual for the 24 months prior to diagnosis date/date of study entry. Exposure levels below the 20th percentile were used as the referent category for each pollutant. Individual monthly air pollutant averages were considered in the 24 months before diagnosis (to explore variations in lag time), but our conclusions did not differ from the 24-month averages; threfore, the results for the 24-month averages are shown.

Potential confounders and effect modifiers were identified through reviewing current literature and by examining causal diagrams to clarify relationships between factors (Rothman et al. 2012). Potential confounders were further assessed using a > 10% change in estimate criteria (Greenland 1989) for several risk factors that could potentially confound the relationship between air pollution and pulmonary TB: cigarette smoking, length of KPNC membership, median household income, percent foreign-born people in the census block, education in the residential census block, alcohol hospitalization, diabetes, and HIV status. Using these criteria, no factors were found to confound the air pollution–pulmonary TB associations and were therefore not included in the final analysis. The final models included only the matching factors (age, sex, and race/ethnicity).

Potential effect modifiers [cigarette smoking, alcohol hospitalizations, diabetes, COPD, HIV status, renal dialysis (all categorized as ever/never), and percent foreign born in census block (continuous)] were further assessed using likelihood ratio tests (LRTs) (Hosmer and Lemeshow 1989). To determine the presence of effect modification, we compared LRTs from models with and without interaction terms included in the conditional logistic regression model. Effect modification was considered present if the LRT differed at a significance level of < 0.10. Only smoking met this criterion, and thus only these stratum-specific ORs are shown.

A multipollutant analysis for air pollution and pulmonary TB association was also considered. All analyses were performed using SAS (version 9.3; SAS Institute Inc., Cary, NC).

## Results

Characteristics of pulmonary TB cases and matched controls nested within the KPNC membership are summarized in Table 1. We identified 2,309 cases of pulmonary TB, and 4,604 controls matched 2:1 to cases by age, sex, and race/ethnicity, which were drawn from the existing KNPC membership during the study period, 1996–2010. Less than one-third (2,028) of the participants were classified as ever smokers. The study population was split equally by sex, although a larger percentage of males were considered ever smokers than females (44.1% vs. 19.9% among cases and 36.0% vs. 20.1% among controls). As expected, never smokers were younger than ever smokers (median = 47 and 54 years, respectively). The proportion of Asians and Hispanics within this study was consistent with the racial/ethnic distribution of residents in this geographic area, as well as the at-risk population for TB in the United States [Centers for Disease Control and Prevention (CDC) 2011].

**Table 1:** Distribution of characteristics of pulmonary tuberculosis disease (TB) cases and matched controls nested within the Kaiser Permanente of Northern California (KPNC) membership, 1996–2010.

Characteristics	All		Ever smokers		Never smokers	
	Cases *n *= 2,309	Controls*n *= 4,604	Cases *n *= 737	Controls*n *= 1,291	Cases*n *= 1,572	Controls*n *= 3,313
ECD demographic factors
Sex
Male	1,144 (49.6%)	2,291 (49.8%)	505 (68.5%)	825 (63.9%)	639 (40.7%)	1,466 (44.3%)
Female	1,165 (50.4%)	2,313 (50.2%)	232 (31.5%)	466 (36.1%)	933 (59.4%)	1,847 (55.6%)
Age
21-34	512 (22.1%)	1,000 (21.7%)	94 (12.8%)	202 (15.7%)	418 (26.6%)	798 (24.1%)
35-49	648 (28.1%)	1,271 (28.1%)	178 (24.1%)	305 (23.6%)	470 (29.9%)	966 (29.2%)
50-64	631 (27.3%)	1,270 (27.6%)	268 (36.4%)	418 (32.3%)	363 (23.1%)	852 (25.7%)
≥ 65	518 (22.4%)	1,063 (23.1%)	197 (26.7%)	366 (28.4%)	321 (20.4%)	697 (21.0%)
Race/ethnicity
Non-Hispanic white	402 (17.4%)	811 (17.6%)	175 (23.7%)	288 (22.3%)	227 (14.4%)	523 (15.8%)
Black	184 (8.0%)	356 (7.7%)	79 (10.7%)	135 (10.5%)	105 (6.7%)	221 (6.7%)
Asian	894 (38.7%)	1,801 (39.1%)	222 (30.1%)	371 (28.7%)	672 (42.7%)	1,430 (43.2%)
Hispanic	451 (19.5%)	912 (19.8%)	142 (19.3%)	263 (20.4%)	309 (19.7%)	649 (19.6%)
Other	150 (6.5%)	298 (6.4%)	56 (7.6%)	124 (9.6%)	94 (6.0%)	174 (0.5%)
Unknown	228 (9.9%)	426 (9.3%)	63 (8.5%)	110 (8.5%)	165 (10.5%)	316 (9.5%)
Length of enrollment
2–5 years	768 (33.3%)	1,493 (32.4%)	220 (29.9%)	378 (29.3%)	548 (34.9%)	1,115 (33.7%)
5–10 years	628 (27.2%)	1,222 (26.5%)	186 (25.2%)	321 (24.9%)	442 (28.1%)	901 (27.2%)
10–15 years	332 (14.4%)	640 (13.9%)	104 (14.1%)	202 (15.7%)	228 (14.5%)	438 (13.2%)
> 15 years	581 (25.2%)	1,249 (27.1%)	227 (30.8%)	390 (30.2%)	354 (22.5%)	859 (25.9%)
Census block demographic factors
Median household income
Median	$60,781	$61,443	$57,361	$59,077	$62,143	$62,566
(IQR)	($46k–$77k)	($46k–78k)	($44k–$72k)	($44k–$76k)	($47k–$79k)	($47k–79k)
Percent foreign born
Median	26.3	21.9	24.4	19.6	27.0	22.8
(IQR)	(15.0–41.0)	(12.5–37.6)	(13.4–40.0)	(11.3–34.2)	(15.7–14.1)	(13.0–38.4)
More than high school education
Median	63	64.2	61.6	63.1	63.2	64.8
(IQR)	(49.4–75.1)	(50.3–76.8)	(48.4–73.2)	(50.0–75.6)	(50.4–75.7)	(50.6–77.1)
ECD medical history
Any tuberculosis risk factors
None	1,292 (56.0%)	3,087 (67.1%)	344 (46.7%)	757 (58.6%)	948 (60.3%)	2,330 (70.3%)
COPD	856 (37.1%)	1,298 (28.2%)	324 (44.0%)	433 (35.5%)	532 (33.8%)	865 (26.1%)
Alcohol hospitalization	107 (4.6%)	169 (3.7%)	84 (11.4%)	98 (7.6%)	23 (1.5%)	71 (2.1%)
Renal dialysis	146 (6.3%)	153 (3.3%)	53 (7.2%)	74 (5.7%)	93 (5.9%)	79 (2.4%)
Immunological prescriptions^*a*^	0	0	0	0	0	0
Diabetes	452 (19.6%)	557 (12.1%)	184 (25.0%)	208 (16.1%)	268 (17.1%)	349 (10.5%)
HIV+	49 (2.1%)	13 (0.3%)	24 (3.3%)	6 (0.5%)	25 (1.6%)	7 (0.2%)
Abbreviations: COPD, chronic obstructive pulmonary disease; ECD, electronic clinical database; HIV+, human immunodeficiency virus positive; IQR, interquartile range.^***a***^Immunological prescriptions include immune-compromising medications known to increase TB risk including glucocorticoids, infliximab, etanercept, adalimumab, certolizumab pegol (Cimzia®), golimumab (Simponi®) and chemotherapy drugs.						

As shown in Table 2, average ambient air pollution concentrations in the 24 months before the diagnosis date varied greatly for participants. Median air pollution concentrations were the same among never smokers and ever smokers for all pollutants, with the exception of CO. Ambient CO concentrations measured in the 24 months prior to pulmonary TB diagnosis/entry into the study were slightly higher for never smokers than for ever smokers. Because the number and location of available monitors within the network varied, particularly for PM_2.5_, which was not routinely monitored until 1999, the number of cases and controls with available pollutant data also varied over the study (see Table 3). As shown in Table 4, Spearman’s correlation air-pollutant averages in the 24 months before the date of diagnosis showed only moderate correlations between pollutants. The strongest correlation observed between ambient averages of air pollutants was seen for PM_10_ and PM_2.5_ (*r* = 0.61).

**Table 2:** Distribution of ambient criteria air pollution concentrations averaged across a 24-month period before the pulmonary tuberculosis (TB) diagnosis/entry into study for cases/matched controls nested within the 1996–2010 KPNC membership with available pollutant monitoring data.

Air pollutant	*n* (%) with pollutant data		Percentile distribution						
	Cases	Controls	Minimum	20th	40th	Median	60th	80th	Maximum
Total population
24-hr PM_2.5_ (μg/m^3^)	1,842 (79.8)	3,661 (79.5)	0.1408	8.5600	9.1840	9.6268	10.3324	11.6602	26.4783
24-hr PM_10_ (μg/m^3^)	2,309 (100.0)	4,604 (100.0)	9.1000	18.3896	19.8733	20.6067	21.6487	24.4700	56.6082
24-hr SO_2_ (ppm)	2,248 (97.4)	4,439 (96.4)	0.0001	0.0009	0.0011	0.0012	0.0013	0.0018	0.0039
24-hr NO_2_ (ppm)	2,309 (100.0)	4,601 (99.9)	0.0003	0.0098	0.0133	0.0144	0.0151	0.0177	0.0390
8-hr O_3_ (ppm)	2,309 (100.0)	4,604 (100.0)	0.0178	0.0279	0.0301	0.0315	0.0330	0.0378	0.0670
8-hr CO (ppm)	2,309 (100.0)	4,598 (99.9)	0.0983	0.5481	0.6793	0.7656	0.8760	1.1114	3.0572
Ever smokers
24-hr PM_2.5_ (μg/m^3^)	617 (83.7)	1,044 (80.9)	0.1408	8.5701	9.2355	9.6085	10.3507	11.7043	21.9646
24-hr PM_10_ (μg/m^3^)	737 (100.0)	1,291 (100.0)	9.1000	18.3547	19.8007	20.5427	21.5647	24.4560	48.6473
24-hr SO_2_ (ppm)	716 (97.2)	1,249 (96.8)	0.0001	0.0008	0.0011	0.0012	0.0013	0.0018	0.0038
24-hr NO_2_ (ppm)	737 (100.0)	1,291 (100.0)	0.0003	0.0100	0.0133	0.0142	0.0149	0.0174	0.0339
8-hr O_3_ (ppm)	737 (100.0)	1,291 (100.0)	0.0178	0.0279	0.0300	0.0315	0.0331	0.0382	0.0670
8-hr CO (ppm)	737 (100.0)	1,289 (99.9)	0.0983	0.5276	0.6628	0.7441	0.8447	1.0882	1.9490
Never smokers
24-hr PM_2.5_ (μg/m^3^)	1,225 (77.9)	2,617 (79.0)	0.1408	8.5589	9.1735	9.6268	10.3206	11.6480	26.4783
24-hr PM_10_ (μg/m^3^)	1,572 (100.0)	3,313 (100.0)	10.3973	18.4381	19.8920	20.6708	21.7093	24.5191	58.6082
24-hr SO_2_ (ppm)	1,532 (97.5)	3,190 (96.3)	0.0000	0.0009	0.0011	0.0012	0.0012	0.0018	0.0039
24-hr NO_2_ (ppm)	1,572 (100.0)	3,310 (99.9)	0.0003	0.0098	0.0134	0.0144	0.0152	0.0178	0.0390
8-hr O_3_ (ppm)	1,572 (100.0)	3,313 (100.0)	0.0182	0.0279	0.0301	0.0315	0.0330	0.0377	0.0670
8-hr CO (ppm)	1,572 (100.0)	3,309 (99.9)	0.2430	0.5537	0.6860	0.7805	0.8877	1.1213	3.0572

**Table 3:** Distance (miles) of residence from closest ambient pollutant monitors for the KPNC cohort, 1996–2010.

Air pollutant	*n*	Mean	Standard deviation	Minimum	Maximum
24-hr PM_2.5_	5,503	7.63	5.91	0.15	48.70
24-hr PM_10_	6,913	6.05	4.24	0.11	35.35
24-hr SO_2_	6,687	14.93	10.74	0.14	49.50
24-hr NO_2_	6,910	6.45	4.78	0.11	45.91
8-hr O_3_	6,913	4.66	3.12	0.05	36.74
8-hr CO	6,907	6.23	4.70	0.05	43.15
KPNC, Kaiser Permanente of Northern California.					

**Table 4:** Spearman correlation coefficients^*a*^ for the estimates of cumulative ambient criteria air pollutant concentrations, 24-month average, among pulmonary tuberculosis (TB) cases and matched controls nested within the 1995–2010 KPNC membership.

Air pollutant	24-hr PM_2.5_	24-hr PM_10_	24-hr SO_2_	24-hr NO_2_	8-hr O_3_	8-hr CO
24-hr PM_2.5_	1	0.61	0.12	0.28	0.25	0.35
24-hr PM_10_		1	0.09	0.33	0.09	0.42
24-hr SO_2_			1	0.19	–0.24	0.30
24-hr NO_2_				1	–0.33	0.23
8-hr O_3_					1	–0.28
8-hr CO						1
^***a***^All coefficients were statistically significant (*p* < 0.05).						

### Pulmonary TB

**Figure 1:**
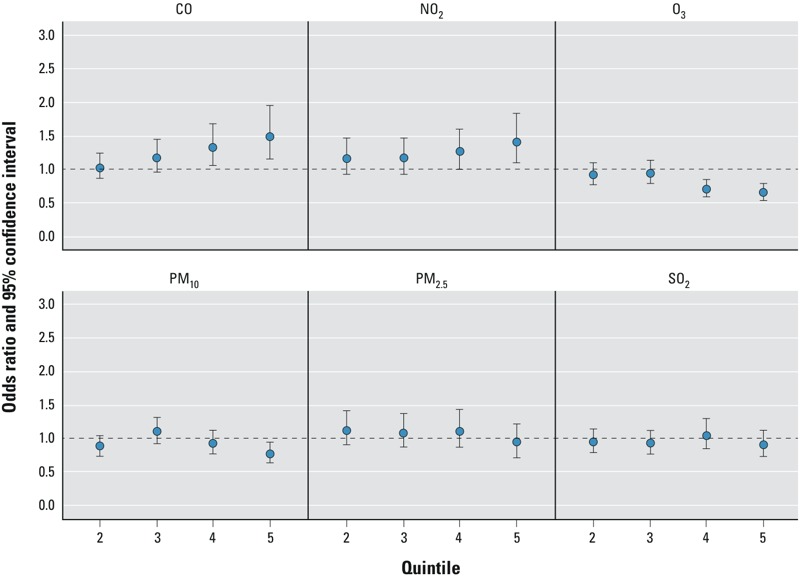
Conditional logistic regression estimated adjusted*^a^* odds ratios (ORs)*^b^* and 95% confidence intervals (CIs) for the associations of pulmonary tuberculosis (pulmonary TB) and quintile*^c^* in the estimates of ambient criteria air pollutant concentrations within the 24-month average before diagnosis or index date, among all cases and matched controls nested within the 1996–2010 KPNC membership. ***^a^***Adjusted for the matching factors (age, sex, and race/ethnicity). ***^b^***ORs relative to the lowest quintile; numeric data provided in Table 4. ***^c^***See Table 2 for quintile cut points.

Figure 1 and Table 5 present the odds ratios (ORs) and 95% confidence intervals (CIs) for the single-pollutant model associations between air pollution (in quintiles) and pulmonary TB in the 24 months prior to diagnosis date/study entry among all cases and matched controls. All effect estimates were adjusted for the matching factors of age, sex, and race/ethnicity. There was no evidence of association between any of the criteria pollutants PM_2.5_, PM_10_, and SO_2_, and pulmonary TB.

**Table 5:** Conditional logistic regression estimated adjusted*^a^* odds ratios (ORs) and 95% confidence intervals (CIs) for associations of pulmonary tuberculosis (pulmonary TB) with ambient criteria air pollutant concentrations, 24-month averages, among all cases and matched controls nested within the 1996–2010 KPNC membership.

Pollutant	Quintile	Single	Multi^*b*^
PM_2.5_	1	Reference	Reference
	2	1.13 (0.90, 1.41)	1.17 (0.92, 1.49)
	3	1.09 (0.87, 1.37)	1.04 (0.84, 1.29)
	4	1.11 (0.87, 1.42)	1.10 (0.87, 1.39)
	5	0.94 (0.73, 1.23)	1.00 (0.83, 1.20)
PM_10_	1	Reference	Reference
	2	0.89 (0.74, 1.06)	0.81 (0.65, 1.00)
	3	1.11 (0.93, 1.32)	1.00 (0.78, 1.28)
	4	0.93 (0.77, 1.11)	0.90 (0.72, 1.13)
	5	0.78 (0.65, 0.94)	0.73 (0.52, 1.01)
SO_2_	1	Reference	Reference
	2	0.95 (0.79, 1.15)	0.91 (0.74, 1.12)
	3	0.93 (0.78, 1.12)	0.89 (0.73, 1.09)
	4	1.05 (0.86, 1.29)	1.03 (0.84, 1.23)
	5	0.90 (0.73, 1.12)	0.91 (0.78, 1.06)
NO_2_	1	Reference	Reference
	2	1.17 (0.93, 1.46)	1.06 (0.81, 1.39)
	3	1.17 (0.93, 1.48)	1.08 (0.80, 1.45)
	4	1.27 (1.01, 1.61)	1.09 (0.79, 1.50)
	5	1.42 (1.10, 1.84)	1.26 (0.85, 1.86)
O_3_	1	Reference	Reference
	2	0.92 (0.78, 1.10)	0.91 (0.73, 1.14)
	3	0.95 (0.80, 1.14)	0.93 (0.74, 1.17)
	4	0.71 (0.59, 0.85)	0.65 (0.51, 0.84)
	5	0.66 (0.55, 0.79)	0.67 (0.49, 0.91)
CO	1	Reference	Reference
	2	1.04 (0.86, 1.24)	0.93 (0.75, 1.16)
	3	1.19 (0.98, 1.45)	1.12 (0.88, 1.43)
	4	1.33 (1.06, 1.68)	1.22 (0.90, 1.66)
	5	1.50 (1.15, 1.95)	1.21 (0.80, 1.83)
^***a***^Adjusted for the matching factors (age, sex, and race/ethnicity). ^***b***^Multi-pollutant model: SO_2_ + PM_10_ + PM_2.5_ + CO + NO_2_ + O_3_.			

As shown in Figure 1 and Table 5, CO and NO_2_ were positively associated with pulmonary TB for the two highest quintiles of exposure in single-pollutant models. For example, the strongest positive effect estimates among all subjects (ever and never smokers combined) for the association of ambient air pollution and pulmonary TB were observed for CO [OR = 1.50 (95% CI: 1.15, 1.95)], as shown in Table 5. Additionally, as measured concentrations of CO exposure increased, the associated odds of pulmonary TB increased. NO_2_ was also positively associated with pulmonary TB, with an apparent dose–response pattern between exposure and pulmonary TB odds.

An inverse association was observed for 8-hr O_3_ and pulmonary TB, with all exposures above the lowest quintile resulting in decreases in the effect estimates, which was evident in the single-pollutant models. For example, compared with those in the lowest quintile of O_3_ exposure, those in the highest quintile had a considerable decrease in risk of pulmonary TB [OR = 0.66 (95% CI: 0.55, 0.79)].

We also considered multi-pollutant models (Table 5). Although the results of the single- and multi-pollutant models were generally consistent, effect estimates were attenuated. For instance, in multi-pollutant models, the OR for the highest quintile of CO exposure was 1.21 (95% CI: 0.80, 1.83). Moreover, the dose-response pattern observed in the single-pollutant model for the association between CO and pulmonary TB became nonmonotonic in the multi-pollutant models. Although the dose–response pattern between NO_2_ exposure and pulmonary TB odds continued to be observed in multi-pollutant models, multi-pollutant CIs included the null value.

### Pulmonary TB Stratified by Smoking

**Figure 2:**
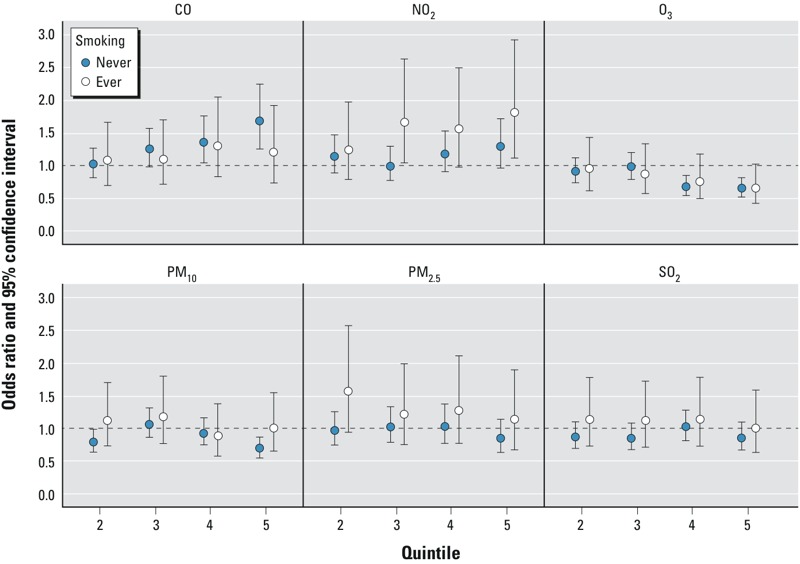
Conditional logistic regression estimated adjusted*^a^* odds ratios (ORs)*^b^* and 95% confidence intervals (CIs) for the associations of pulmonary tuberculosis (pulmonary TB) and quintile*^c^* in the estimates of ambient criteria air pollutant concentrations within the 24-month average before diagnosis or index date, among cases and matched controls nested within the 1996–2010 KPNC membership stratified by smoking status (ever vs. never smokers). ***^a^***Adjusted for the matching factors (age, sex, and race/ethnicity). ***^b^***ORs relative to the lowest quintile; numeric data provided in Table 4. ***^c^***See Table 2 for quintile cut points.

In single-pollutant models, cigarette smoking was found to be an effect modifier of the association between ambient CO and pulmonary TB (*p* = 0.10) for the interaction on a multiplicative scale. Following stratification, a dose–response pattern persisted among never smokers, and the effect estimates for the highest quintiles of 8-hr CO exposure, compared with those of the lowest, were more pronounced among never smokers [OR = 1.68 (95% CI: 1.26, 2.24)] than among ever smokers [OR = 1.19 (95% CI: 0.74, 1.92)], as shown in Figure 2 and Table 6.

**Table 6:** Conditional logistic regression estimated adjusted^*a*^ odds ratios (ORs) and 95% confidence intervals (CIs) for associations of pulmonary tuberculosis (pulmonary TB) with ambient criteria air pollutant concentrations, 24-month averages, among cases and matched controls nested within the 1996–2010 KPNC membership, stratified by smoking status.

Pollutant	Quintile	Never smokers	Ever smokers	*p*-Value^*b*^
PM_2.5_	1	Reference	Reference
	2	0.98 (0.75, 1.27)	1.57 (0.95, 2.58)
	3	1.03 (0.79, 1.34)	1.22 (0.75, 2.00)
	4	1.04 (0.78, 1.37)	1.28 (0.78, 2.11)
	5	0.85 (0.64, 1.15)	1.13 (0.68, 1.89)	0.2812
PM_10_	1	Reference	Reference
	2	0.80 (0.65, 0.99)	1.12 (0.73, 1.70)
	3	1.07 (0.87, 1.32)	1.19 (0.78, 1.80)
	4	0.94 (0.76, 1.16)	0.90 (0.59, 1.37)
	5	0.69 (0.55, 0.87)	1.01 (0.66, 1.56)	0.0854
SO_2_	1	Reference	Reference
	2	0.88 (0.70, 1.10)	1.15 (0.74, 1.79)
	3	0.86 (0.69, 1.08)	1.11 (0.72, 1.73)
	4	1.02 (0.80, 1.29)	1.14 (0.73, 1.79)
	5	0.86 (0.67, 1.10)	1.01 (0.64, 1.58)	0.6422
NO_2_	1	Reference	Reference
	2	1.15 (0.89, 1.48)	1.24 (0.79, 1.97)
	3	1.00 (0.77, 1.29)	1.66 (1.05, 2.63)
	4	1.18 (0.91, 1.53)	1.57 (0.99, 2.49)
	5	1.29 (0.97, 1.71)	1.81 (1.13, 2.92)	0.1860
O_3_	1	Reference	Reference
	2	0.91 (0.74, 1.12)	0.94 (0.62, 1.43)
	3	0.98 (0.80, 1.21)	0.88 (0.58, 1.33)
	4	0.68 (0.55, 0.85)	0.76 (0.50, 1.17)
	5	0.65 (0.52, 0.81)	0.66 (0.43, 1.02)	0.8170
CO	1	Reference	Reference
	2	1.02 (0.82, 1.27)	1.08 (0.70, 1.66)
	3	1.25 (0.99, 1.58)	1.10 (0.71, 1.70)	
	4	1.36 (1.05, 1.77)	1.30 (0.83, 2.05)
	5	1.68 (1.26, 2.24)	1.19 (0.74, 1.92)	0.0979
^***a***^Adjusted for the matching factors (age, sex, and race/ethnicity). ^***b***^*p*-Value of likelihood ratio test comparing model fit with and without inclusion of interaction terms.				

Once stratified on smoking status, the dose–response pattern previously observed in nonstratified models was less pronounced. The association between NO_2_ and pulmonary TB, in the single-pollutant models, was more pronounced among ever smokers [OR = 1.81 (1.13, 2.92) for the highest vs. the lowest quintile of exposure] than among never smokers [OR = 1.29 (0.97, 1.71)], but based on LRT heterogeneity in the effect estimates was not statistically significant (*p* = 0.19).

Upon stratification by smoking status, the strength of the inverse association was similar among both never smokers and ever smokers in the single-pollutant models for the association between O_3_ and pulmonary TB.

Stratified analyses of the multi-pollutant model estimates by cigarette smoking status yielded results similar to those of the single-pollutant analysis (see Table S1).

## Discussion

In this sample of residents of northern California (2,309 cases and 4,604 controls), which is, to the best of our knowledge, the first large U.S. study within a well-defined population to assess the potential associations between individual-level estimates of the criteria air pollutants and pulmonary TB, exposure to average 8-hr CO concentrations in the highest quintile during the 24 months before diagnosis was associated with a 50% elevation in the odds of developing pulmonary TB (95% CI: 15, 95%) relative to the odds among those in the lowest quintile of exposure. When the analysis was stratified by smoking status, effect estimates for the association between CO and pulmonary TB were slightly stronger for never smokers and weaker or null for ever smokers. Positive associations were also noted for NO_2_, although the magnitude of the association was stronger in ever smokers than in never smokers. In addition, effect estimates were reduced by approximately one-third for O_3._ No consistent associations were observed among the other pollutants studied, including PM_2.5_, PM_10_, and SO_2_.

Multi-pollutant analyses were conducted to assess the association of combined exposure to several air pollutants with odds of pulmonary TB. Although the multi-pollutant model may account for possible mutual confounding between pollutants, there is the potential for variance inflation and bias in analyses that adjust for multiple pollutants if these are highly correlated (Rothman et al. 2012; Schisterman et al. 2009). Because it is unclear which model resulted in the least-biased estimates, both single- and multi-pollutant model results were presented. Several of the effect estimates from the multi-pollutant models for the association between air pollution and pulmonary TB are attenuated toward the null value, with wider confidence intervals than in the single-pollutant model; however, the effect estimates continue to reflect the same patterns of association in general. These results emphasize the need for precise effect estimates as well as for improved assessment of both air pollution exposure concentrations and smoking status.

The associations observed for CO were consistent with other available evidence on this issue. Exposure to ambient CO in the United States in this setting is primarily a marker of exposure to combustion products and secondary vehicular traffic (U.S. EPA 2010), and experimental studies have shown that diesel exhaust affects immune processes that inhibit TB in mice (Hiramatsu et al. 2005; Saito et al. 2002b). The findings of a previous study of combustion products are consistent with the hypothesis that coal consumption, according to annual historical statistics, may be linked to TB because both have followed similar trends in the United States, Canada, and China (Tremblay 2007), although this is a crude ecological comparison (Tremblay 2007).

In our study, risk of pulmonary TB was also positively associated with exposure to NO_2_. This gaseous pollutant is produced primarily from combustion sources, such as motor vehicle exhaust and electric generating units (U.S. EPA 2008a). Individuals living near busy roads are therefore more likely to be exposed to NO_2_ pollution (Gilbert et al. 2007; U.S. EPA 2008a) than individuals who do not, and they may disproportionately suffer from any related health effects [U.S. EPA 2008b; Health Effects Institute Panel on the Health Effects of Traffic-Related Air Pollution (HEI) 2010; Oosterlee et al. 1996]. Although individual pollutant exposure depends predominantly on local outdoor concentrations, indoor pollution such as smoking and using gas appliances may alter exposure levels (U.S. EPA 2008b). It is also possible that the associations with NO_2_ may represent exposure to traffic-related air pollution rather than exposure to NO_2_ specifically.

In this study, an inverse association was observed for O_3_ and pulmonary TB, with all exposures above the lowest quintile resulting in decreases in pulmonary TB risk. This observed relationship between O_3_ concentrations and pulmonary TB is not entirely unexpected because levels of ambient O_3_ and NO_2_ are bonded by chemical coupling. In the presence of sunlight, nitrogen is depleted in the formation of O_3_ (Clapp and Jenkin 2001; U.S. EPA 2013). As a result, individuals exposed to high levels of NO_2_ often experience low levels of O_3_, particularly along high-traffic areas (Jerrett et al. 2005; Lipsett et al. 2011).

We observed no consistent association between PM_2.5_ and pulmonary TB. We are aware of one previous study (Jassal et al. 2013) that reported a positive association between ambient PM_2.5_ and the odds of having acid fast bacilli detected (vs. not detected) on a sputum smear (an indication of active TB); however, this finding was based on a population of fewer than 200 hospital patients in Los Angeles, California. In contrast, our results are based on nested case–control study data for 6,913 participants who were broadly representative of the northern California population from which they were drawn, and our cases were compared with a control population that did not have active TB. Additional studies are needed to elucidate possible associations between PM_2.5_ and active pulmonary TB.

Assigning individual average exposures by relying on the ambient air monitoring near to the participant’s home likely introduces some exposure misclassification. This issue may be particularly relevant to CO assessments because of considerable spatial variability in ambient CO concentrations (U.S. EPA 2010). Further, exposure misclassification might have occurred as a result of variations in residential ventilation systems, levels of physical activity, or time spent outdoors, away from home, or traveling by car outside of the participant’s residential area. This last item is important because considerable CO exposure can be experienced while driving or riding in vehicles (U.S. EPA 2010). However, these potential sources of error are common to many epidemiologic studies focused on assessing health effects associated with air pollution that have also used our approach to estimate individual-level ambient concentrations (Brauer et al. 2008; Brunekreef et al. 2009; Lipsett et al. 2011). These errors are perhaps nondifferential, given that exposure estimates for our cohort of Kaiser subjects were constructed at CARB (by C.G.), without knowledge of the subjects’ outcome status, by matching the subject address recorded in each subject’s medical record with the air pollutant concentrations recorded at the nearest monitor. In addition, cases and controls were nested in the Kaiser cohort and were required to have at least 2 years of Kaiser membership for study eligibility, and we adjusted for census-block–derived education and income, all of which would help to reduce any differential misclassification bias associated with residential location and hence exposure estimation. All of these issues would increase the likelihood of nondifferential exposure misclassification, which may bias results toward the null (Rothman et al. 2012). However, differential misclassification is possible, given that assignment of exposure status was not dichotomous; thus, spurious associations could arise. Perhaps by improving exposure estimation through the use of sophisticated methods such as land use regression or kriging (Hoek et al. 2008; Jerrett et al. 2005), future studies could enhance our ability to determine the true magnitude of the air pollution–TB association.

To examine the potential role of smoking in the association between air pollution and TB, participants were considered never smokers if there was no indication of smoking anywhere in their records. This method of categorizing smokers may have resulted in underestimation of smoking prevalence in this study population if smoking status was underreported in the clinical data. This underestimation could have lead to a spurious result when smoking status was included in the analyses if the error was differential by air pollution exposure status. However, the assignment of smoking status was made blinded to case status and air pollution exposure status; thus, differential misclassification of smoking seems unlikely. Further, for study eligibility, cases and controls were matched on age and were required to have Kaiser membership of at least 2 years in duration, both of which would help to reduce—although not entirely eliminate—the likelihood of differential exposure misclassification because of cohort or acculturation differences. Nondifferential error of a covariate would result in attenuation of the effect estimates (Rothman et al. 2012).

Recent immigration status, a factor that puts individuals at disproportionately increased risk of TB in the United States (CDC 2011), was not available for cases and controls in this population. Instead, we included the percent of the census block that was foreign-born in our early statistical models; however, the covariate did not confound our associations. Although use of this proxy is inexact, mandatory screening confirms that all immigrants should be free of active TB upon entry into the United States (Liu et al. 2009). Furthermore, to ensure that recent immigrants (those at the highest risk) were screened out of the study and that we captured cases of TB that were activated within the United States, we implemented a 2-year KPNC membership requirement for study eligibility.

This nested case–control study conducted within a large well-defined population has several advantages. The KPNC membership provided a clear sampling framework for selection of controls and enabled a broad representation of the population at large. However, KPNC members were somewhat better educated than the surrounding geographic population, had fewer individuals in the income extremes, and had a lower prevalence of smokers (Gordon 2006). Nevertheless, the uniform access to health care in this population minimized the potential for selection bias, and the availability of detailed clinical data allowed us to explore potential confounding and effect modification of numerous risk factors associated with TB including chronic obstructive pulmonary disease (COPD), alcohol hospitalizations, diabetes, renal dialysis, and immunosuppressive conditions including HIV (CDC 2011; Liu et al. 2009). Finally, the availability of the criteria air pollution data permitted us to construct ambient exposure concentrations during the 24-month period prior to a diagnosis of pulmonary TB for all KPNC study participants with available pollutant data; this period is considered the critical window between exposure to mycobacteria and development of active pulmonary TB (Raviglione et al. 1995; WHO 2010).

The persistence of ambient air pollution remains a major public health problem, and millions worldwide die each year from causes directly related to air pollution (WHO 2011). Although levels of air pollution have continually dropped in the United States and in other developed countries in recent years, levels in developing countries remain high and are even increasing in many areas (WHO 2011). Many of the same countries with high levels of air pollution are also burdened with the highest levels of TB and increasing prevalence of cigarette smoking (Cohen and Mehta 2007; Hassmiller 2006).

## Conclusion

In conclusion, our results showed positive associations between ambient concentrations of CO and NO_2_ and risk of pulmonary TB among residents of northern California. To our knowledge, this is the first large, nested case–control study with individual-level estimates of air pollution concentrations that has been conducted in the United States; thus, future studies in places similar to the United States (i.e., with low TB rates and low air pollution levels) using larger sample sizes and improved characterization of smoking status (both allowing for examination within various smoking strata) are needed to confirm our findings. Studies are also needed in countries outside the United States that experience higher rates of TB and increased exposure to outdoor air pollution. Given the large number of people worldwide who are infected with *Mycobacterium tuberculosis* and are exposed to high concentrations of air pollution, any association between air pollution and TB is of considerable public health importance because attention to the impacts of air quality may contribute to global TB control.

## Supplemental Material

(1.9 MB) PDF
