# Supplementary material for: Air Pollution and Pulmonary Tuberculosis: A Nested Case–Control Study among Members of a Northern California Health Plan
Source: Environ Health Perspect. 2016 Feb 9;124(6):761–8. doi: 10.1289/ehp.1408166 (PMC4892908; doi:10.1289/ehp.1408166)

Environ Health Perspect
DOI: 10.1289/ehp.1408166

Note to readers with disabilities: *EHP* strives to ensure that all journal content is accessible to all readers. However, some figures and Supplemental Material published in *EHP* articles may not conform to 508 standards due to the complexity of the information being presented. If you need assistance accessing journal content, please contact ehp508@niehs.nih.gov. Our staff will work with you to assess and meet your accessibility needs within 3 working days.

Supplemental Material

Air Pollution and Pulmonary Tuberculosis: A Nested Case-Control Study among Members of a Northern California Health Plan

Geneé S. Smith, Stephen K. Van Den Eeden, Cynthia Garcia, Jun Shan, Roger Baxter, Amy H. Herring, David B. Richardson, Annelies Van Rie, Michael Emch, and Marilie D. Gammon

Table of Contents

Table S1. Multi-pollutant conditional logistic regression estimated adjusted odds ratios (ORs) and 95% confidence intervals (CIs) for associations of pulmonary tuberculosis and ambient criteria air pollutants concentrations, 24-month averages, among cases and matched controls nested within the 1996-2010 KPNC membership, stratified by smoking status.

Figure S1. Available $PM_{2.5}$ pollutant monitors in California

Figure S2. Available PM_{10} pollutant monitors in California

Figure S3. Available SO_2 pollutant monitors in California

Figure S4. Available NO_2 pollutant monitors in California

Figure S5. Available O_3 pollutant monitors in California

Figure S6. Available CO pollutant monitors in California

Table S1. Multi-pollutant[a] conditional logistic regression estimated adjusted[b] odds ratios (ORs) and 95% confidence intervals (CIs) for associations of pulmonary tuberculosis and ambient criteria air pollutants concentrations, 24-month averages, among cases and matched controls nested within the 1996-2010 KPNC membership, stratified by smoking status.

**Pollutant**	**Quintile**	**Never Smokers**	**Ever Smokers**
**$PM_{2.5}$**	1	ref	ref
	2	1.17 (0.94, 1.46)	1.72 (0.84, 3.53)
	3	1.09 (0.84, 1.42)	1.37 (0.65, 2.89)
	4	1.17 (0.85, 1.62)	1.83 (0.73, 4.59)
	5	0.99 (0.80, 1.23)	1.35 (0.48, 3.78)
**PM_{10}**	1	ref	ref
	2	0.79 (0.62, 1.01)	0.91 (0.52, 1.59)
	3	1.01 (0.78, 1.31)	1.16 (0.58, 2.32)
	4	0.84 (0.63, 1.12)	0.86 (0.37, 1.99)
	5	0.71 (0.50, 1.01)	1.10 (0.45, 2.69)
**SO_2**	1	ref	ref
	2	0.93 (0.74, 1.17)	1.13 (0.73, 1.75)
	3	0.76 (0.60, 0.96)	1.10 (0.68, 1.78)
	4	1.10 (0.83, 1.47)	1.10 (0.67, 1.81)
	5	0.78 (0.62, 0.97)	0.97 (0.51, 1.81)
**NO_2**	1	ref	ref
	2	1.04 (0.80, 1.35)	1.19 (0.57, 2.48)
	3	1.07 (0.81, 1.41)	1.35 (0.61, 2.99)
	4	1.08 (0.80, 1.46)	1.57 (0.62, 3.99)
	5	1.27 (0.84, 1.92)	1.75 (0.82, 3.72)
**O_3**	1	ref	ref
	2	0.96 (0.57, 1.62)	0.95 (0.52, 1.73)
	3	0.76 (0.42, 1.40)	0.98 (0.52, 1.85)
	4	0.78 (0.41, 1.45)	0.68 (0.35, 1.31)
	5	0.76 (0.39, 1.49)	0.97 (0.42, 2.20)
**CO**	1	ref	ref
	2	1.15 (0.97, 1.36)	1.04 (0.53, 2.04)
	3	1.33 (1.02, 1.73)	1.16 (0.68, 1.99)
	4	1.57 (1.12, 2.20)	1.35 (0.57, 3.20)
	5	1.55 (1.23, 1.95)	1.29 (0.56, 2.97)

[a] Multi-Pollutant Model: SO_2+PM_{10}+$PM_{2.5}$+CO+NO_2+ O_3

[b]Adjusted for the matching factors (age, gender, and race/ethnicity).

Figure S1. Available $PM_{2.5}$ pollutant monitors in California

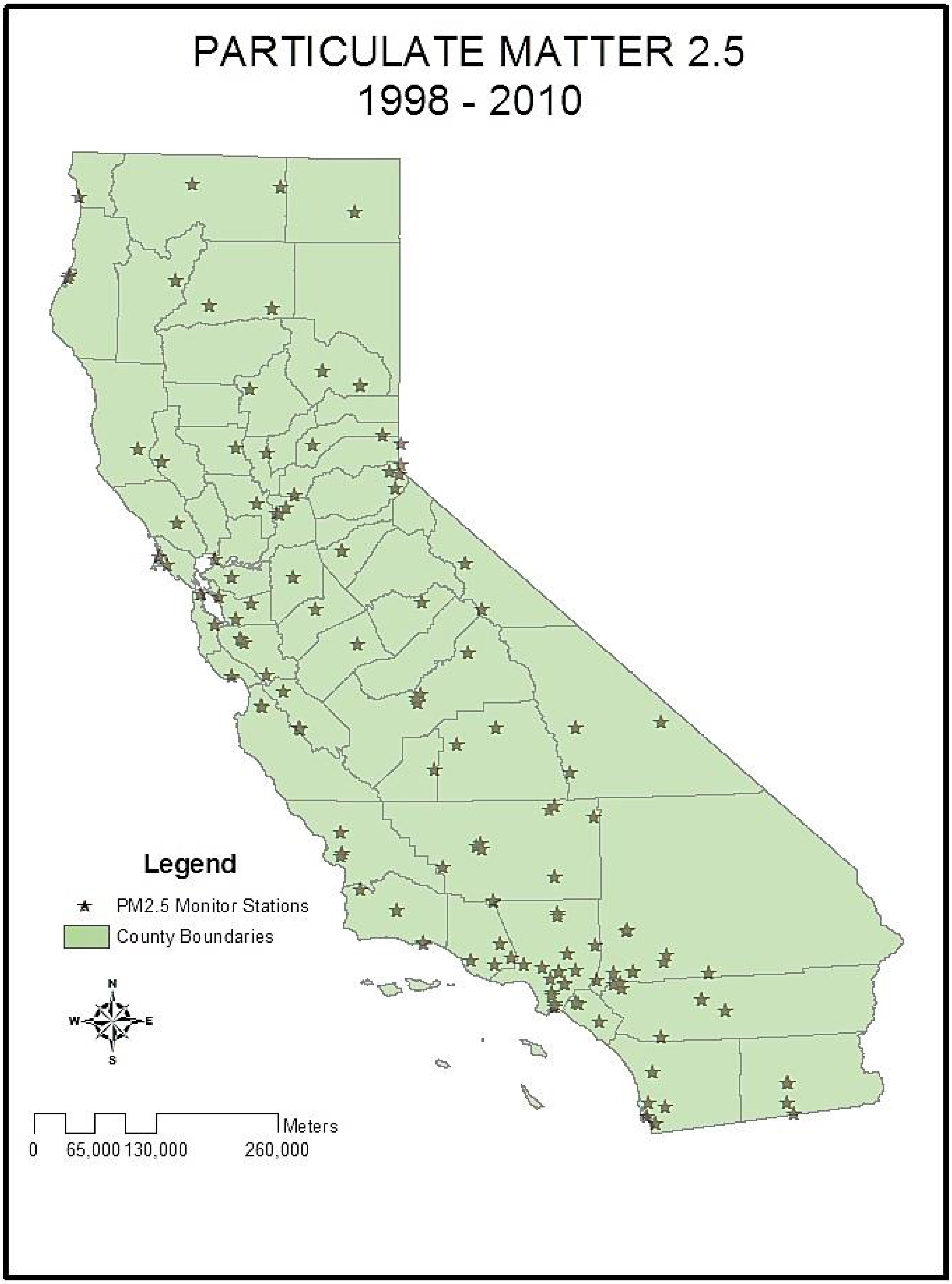

Figure S2. Available PM_{10} pollutant monitors in California

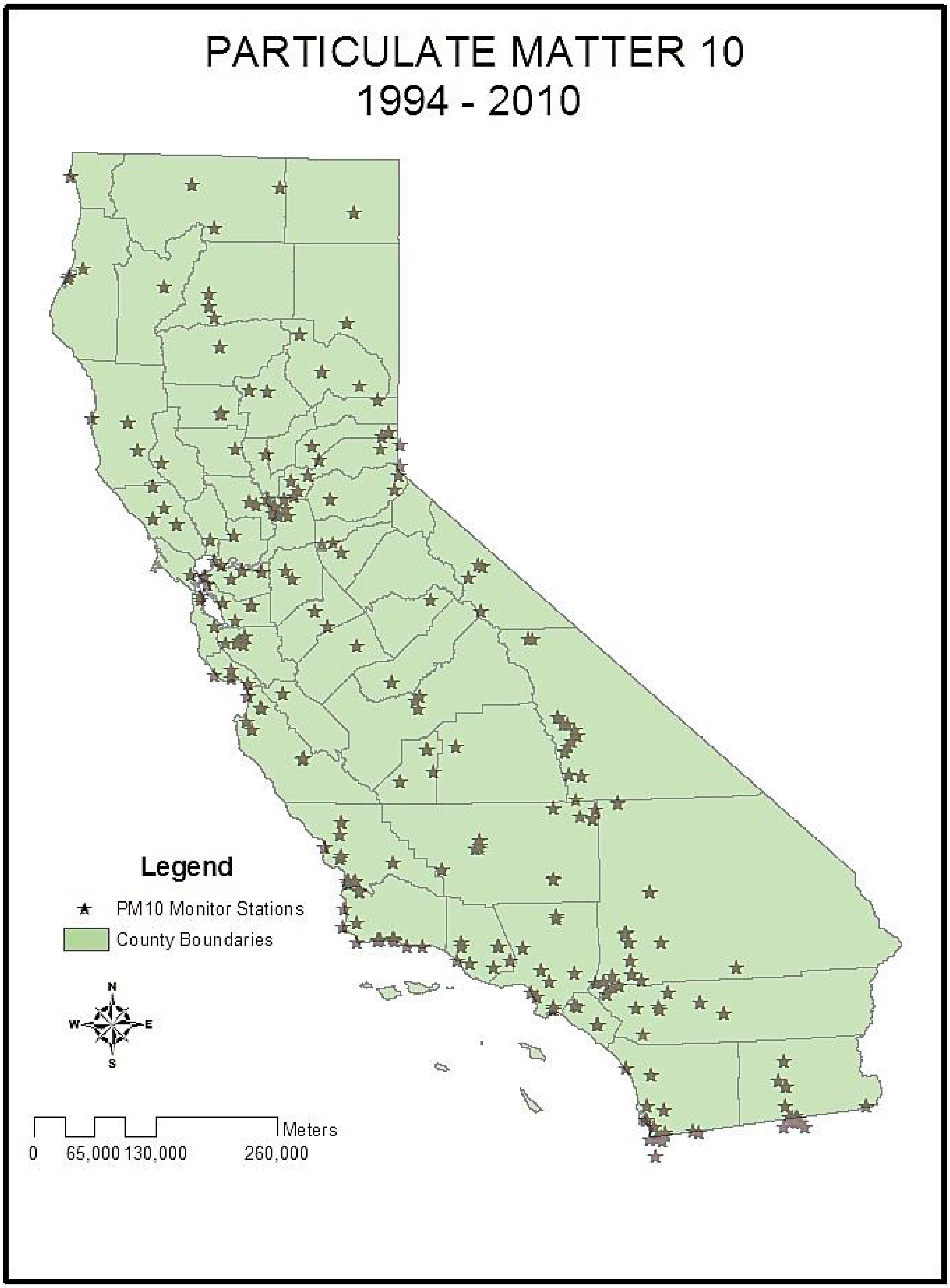

Figure S3. Available SO_2 pollutant monitors in California

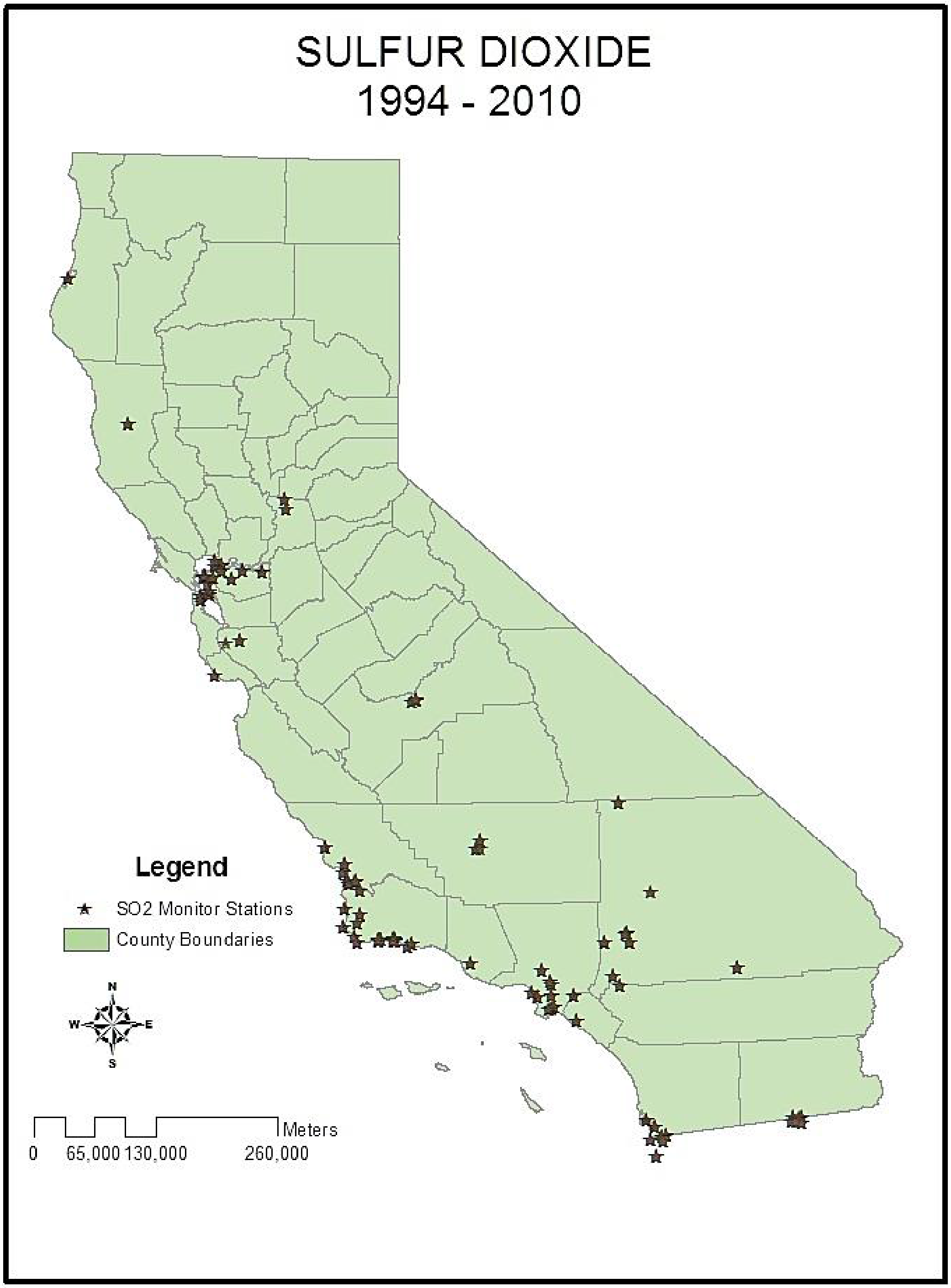

Figure S4. Available NO_2 pollutant monitors in California

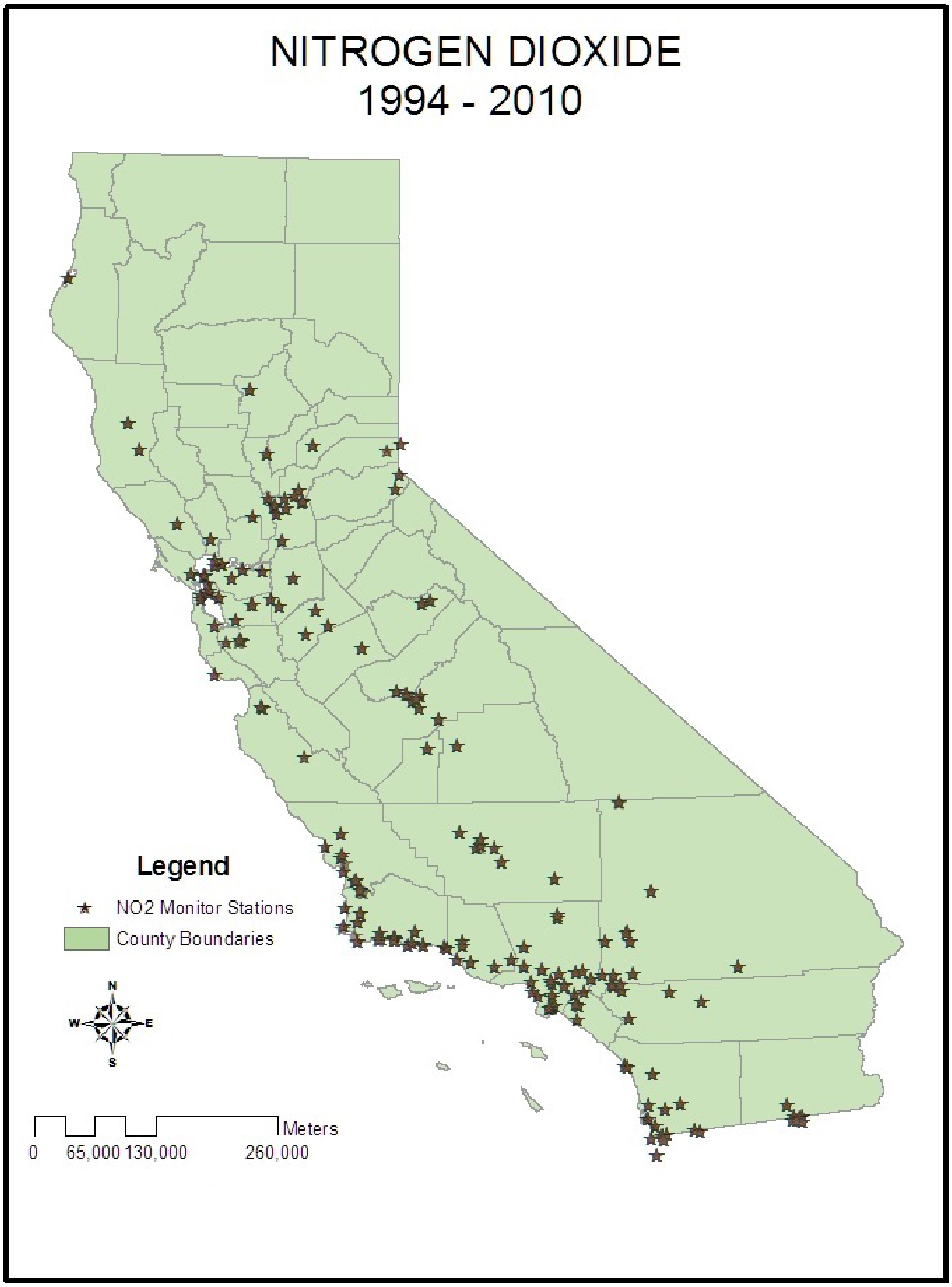

Figure S5. Available O_3 pollutant monitors in California

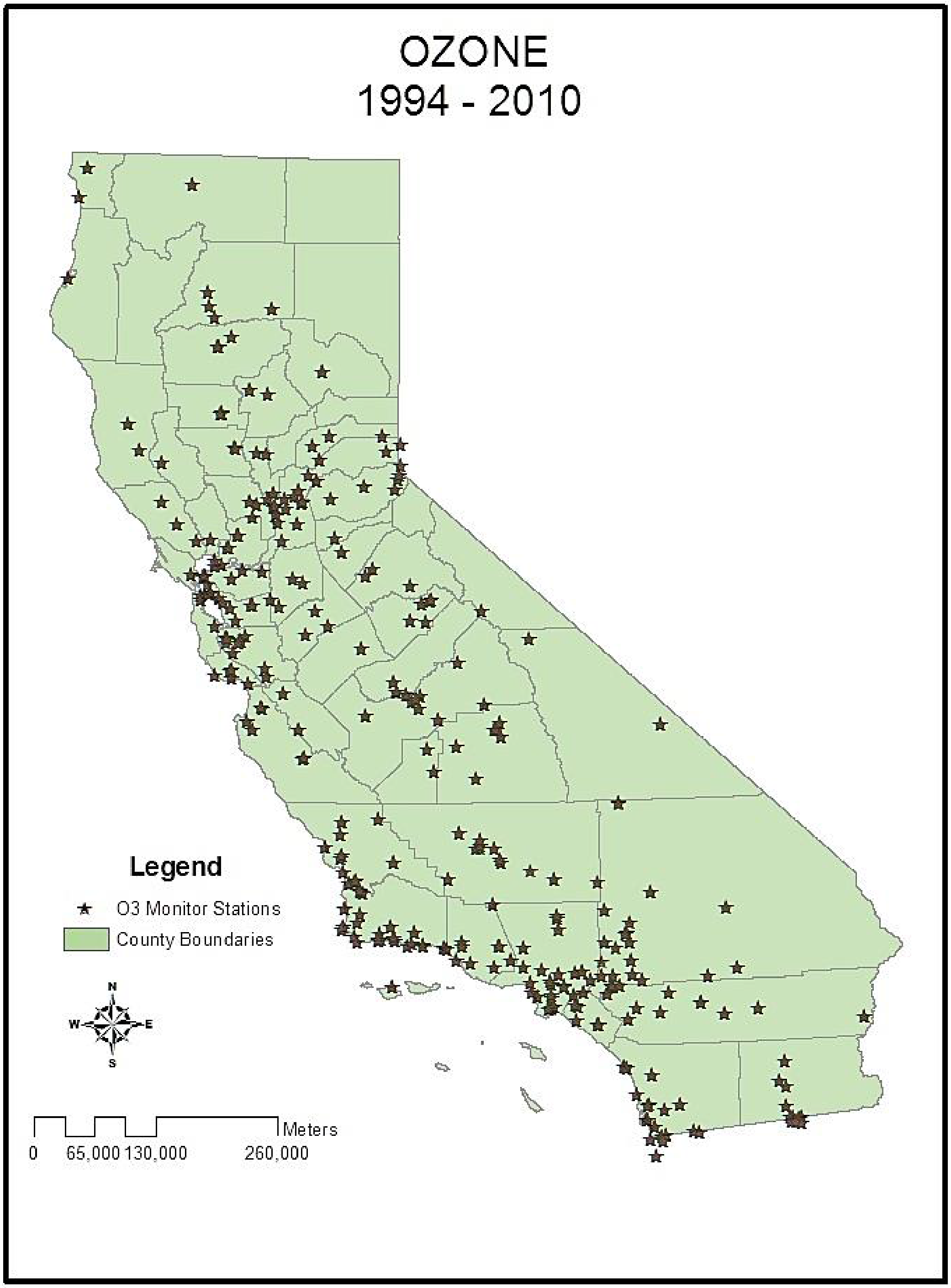

Figure S6. Available CO pollutant monitors in California

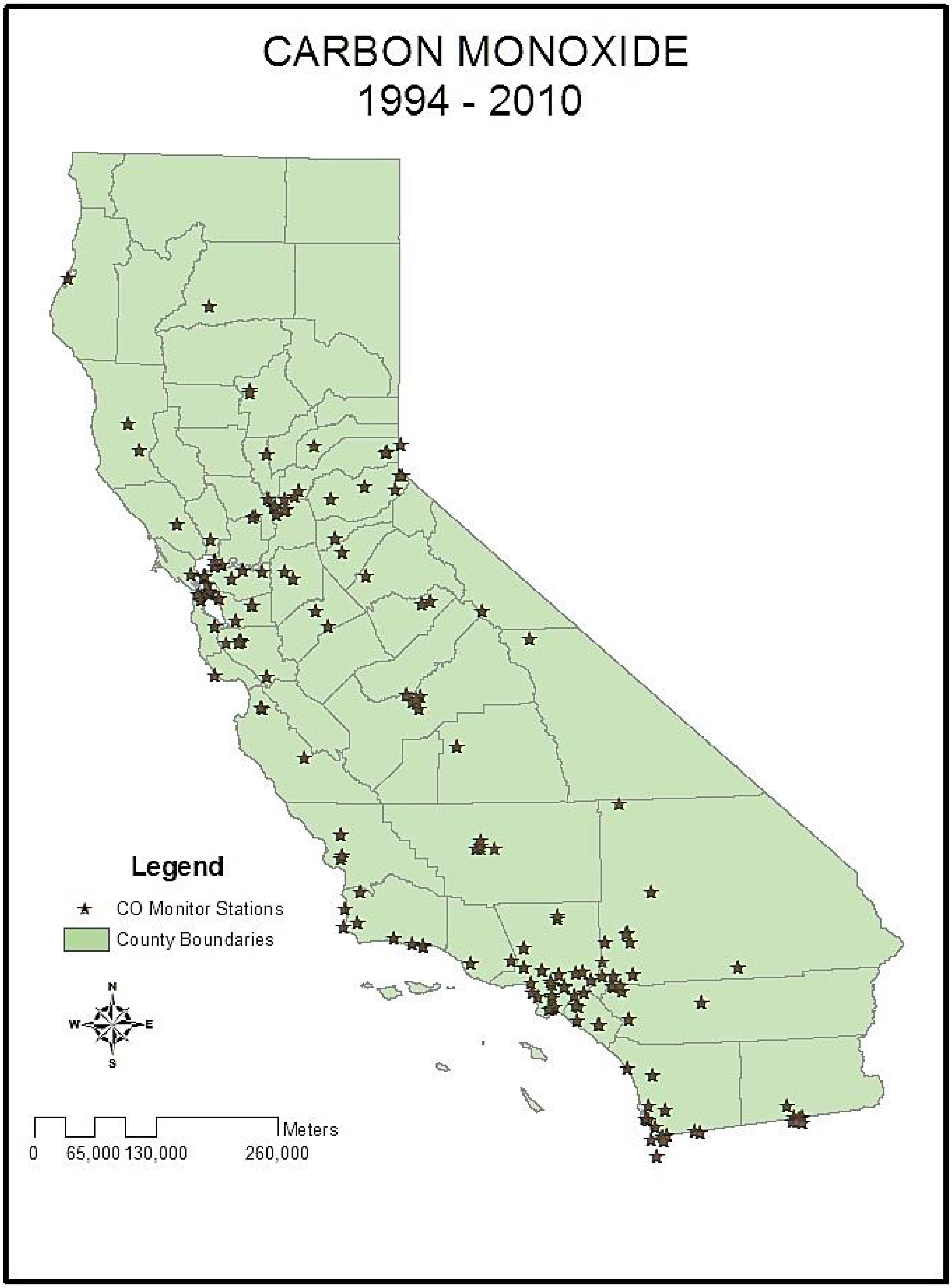

Supplement: (1.9 MB) PDF [file ehp.1408166.s001.acco.pdf]
